# Adaptive Learning Emotion Identification Method of Short Texts for Online Medical Knowledge Sharing Community

**DOI:** 10.1155/2019/1604392

**Published:** 2019-06-25

**Authors:** Dan Gan, Jiang Shen, Man Xu

**Affiliations:** ^1^College of Management and Economics, Tianjin University, Tianjin 300072, China; ^2^Business School, Nankai University, Tianjin 300071, China

## Abstract

The medical knowledge sharing community provides users with an open platform for accessing medical resources and sharing medical knowledge, treatment experience, and emotions. Compared with the recipients of general commodities, the recipients in the medical knowledge sharing community pay more attention to the intensity or overall evaluation of emotional vocabularies in the comments, such as treatment effects, prices, service attitudes, and other aspects. Therefore, the overall evaluation is not a key factor in medical service comments, but the semantics of the emotional polarity is the key to affect recipients of the medical information. In this paper, we propose an adaptive learning emotion identification method (ALEIM) based on mutual information feature weight, which captures the correlation and redundancy of features. In order to evaluate the proposed method's effectiveness, we use four basic corpus libraries crawled from the Haodf's online platform and employ Taiwan University NTUSD Simplified Chinese Emotion Dictionary for emotion classification. The experimental results show that our proposed ALEIM method has a better performance for the identification of the low-frequency words' redundant features in comments of the online medical knowledge sharing community.

## 1. Introduction

More and more comments, opinions, suggestions, ratings, and feedback are produced on social networks with the rapid development of the Internet [1]. While those on social networks are meant to be useful, this part of the contents requires adopting text mining and emotion analysis techniques. Until now, emotional analysis and evaluation still face several challenges [2], which are shown in Table 1. These challenges become obstacles to accurately analyze emotional polarity.

In recent years, more and more research has been done on emotion analysis. Among them, unstructured natural language texts have received the widest attention of scholars [9]. Emotion analysis is the inference of users' opinions, positions, and attitudes through written or spoken contents [10]. Solving emotion analysis tasks typically uses dictionary-based and learning-based approach [11, 12]. The dictionary-based approach analyzes the relevance of each word to a particular emotion by using the predefined dictionary [13]. Learning-based methods typically use labeled samples to train the specific-purpose models under supervision [14].

Emotional analysis is increasingly used to analyze human emotions, but the fatal shortcoming of current emotion analysis methods is lack of aspect level granularity improvement, and also they are rarely applied to online knowledge communities, especially medical knowledge communities, so it is necessary to find an emotional classification method for medical knowledge communities. In light of these considerations, we propose an adaptive learning emotion identification method (ALEIM) based on mutual information feature weight, which captures the correlation and redundancy of features. Its effectiveness is verified on the datasets crawled from the Haodf's online platform, in which the eigenvalues corresponding to the feature nouns are assigned according to the emotional dictionary NTUSD compiled by Taiwan University. Finally, the experimental results show that our proposed ALEIM method achieves a better performance.

The remainder of this paper is organized as follows.  Section 2 reviews the related work of our study.  Section 3 presents our proposed ALEIM method, which contains problem description and assumptions, feature selection based on mutual information, and emotional polarity selection based on mutual information weight.  Section 4 presents the datasets, evaluation measures, experimental performance, and the discussion. Finally, Section 5 presents the conclusions.

## 2. Related Work

### 2.1. Feature Extraction

Natural language processing and text analysis techniques are used to extract emotion features in emotion comments [9]. However, the feature selection method based on mutual information is developed to obtain the true feature, which is an information entropy estimation method independent of classifiers and datasets and superior to other feature extraction methods [15, 16]. A redundant algorithm for constructing the mutual information feature subset was proposed and used to improve the emotion classification accuracy [17]. The maximal relevance and minimal redundancy (mRMR) algorithm was proposed on the basis of the principle of mutual information, which was compared with the SVM classification [18, 19] and the recommended three ratio classification methods; the proposed accuracy is superior to traditional method, and recognition speed is faster than the intelligent method [20].

### 2.2. Emotion Analysis

Emotion analysis has been widely used in many fields [21, 22], such as consumer management, precision marketing, social network, etc. Unsupervised learning algorithm and the foremost supervised learning algorithm were used to classify emotion polarity of comments [23]. Moreover, emotion analysis is divided into many levels: document level [24], sentence level [25], word/term level, or aspect level [26].

Until now, the emotion classification methods can be roughly divided into three fields: machine learning methods, emotion dictionary-based methods [27], and deep learning emotion classification approaches [28]. Some common classifiers for machine learning methods are decision trees [29], Bayes [30], and support vector machines [31]. Emotion dictionary-based approach is to achieve classification by using the different granularity of emotion words polarity. The common emotion lexicons include the following: SentiWordNet [32], General Inquirer [33], SenticNet [34], Opinion Lexicon, HowNet Emotional Dictionary, Subjective Lexicon, DUTIR emotional vocabulary ontology library, and NTUSD [35]. However, it is very difficult to construct a complete emotion dictionary, which may have polarity of all emotion words. Therefore, it is necessary to obtain the polarity of emotional words by context. Deep learning emotion classification approaches are usually used to achieve emotion classification at aspect level. In terms of natural language processing, deep learning has far superior performance to machine learning [18], and it has been proved in the fields of text recognition [36] and semantic mining [37]. More recently, deep learning, especially convolutional neural network is widely used to improve the emotion analysis accuracy [38–40].

## 3. Semisupervised Emotion Classification Method

### 3.1. Problem Description and Assumptions

Let the basic corpus denote Θ=(*U*, *A*, *V*, *f*), the domain *U* indicate the source review set exists *N* comments, *U*={*u*_*i*_ | *i*=1,2,…, *N*}, *u*_*i*_ be the *n*th comment, and *N* be the total number of comments. The feature noun set of comments denotes *A*={*a*_*j*_ | *j*=1,2,…, *J*}, *a*_*j*_ is the *j*th comment feature, and *J* is the total number of feature noun. Among them, the overall characteristics of the review (patient satisfaction, efficacy) are also known as the identification category, which is recorded as *c*. The range of eigenvalues is *V*; it forms an information function with *U* and *A*: *f* : *U* × *A*⟶*V*. Let *V*={*V*^(*u*_*i*_)^ | *u*_*i*_ ∈ *U*}; then, *V*^(*u*_*i*_)^ is the eigenvalue vector of the comment *u*_*i*_ and *V*^(*u*_*i*_)^={*V*_*ij*_ | *j*=1,2, ..., *k*,  *j* ≤ *k*}, and *k* is the number of eigenvalues of the feature noun *a*_*i*_. *V*_*ij*_ is the *j*th eigenvalue of the *u*_*i*_ comment (the eigenvalue is related to the adjective corresponding to the noun). The new comment is recorded as *T*; the comment feature matrix can be defined as *V*=[*V*_U_ … *V*_T_]^*T*^. The data in the comments are multiisomerized, so it is necessary to normalize the eigenvalues.

(1) $$\begin{array}{c} v_{j}^{\left(u_{i}\right)}=\frac{V_{ij}-\min_{i}V_{ij}}{\max_{i}V_{ij}-\min_{i}V_{ij}}. \end{array}$$

We number all the adjectives contained in each feature and substitute the number as eigenvalues into the matrix for subsequent calculations.

Let *M*(*c*; *f*_*λ*_) be the *j*th eigenvalues of the comment *u*_*i*_; then, *V* is converted to *V*^*∗*^.

(2) $$\begin{array}{c} V^{*}=\left[\frac{V_{\text{U}}}{V_{\text{T}}}\right]=\left[\begin{array}{cccc} v_{1}^{\left(u_{1}\right)} & v_{2}^{\left(u_{1}\right)} & \cdots & v_{k}^{\left(u_{1}\right)}\\ v_{1}^{\left(u_{2}\right)} & v_{2}^{\left(u_{2}\right)} & \cdots & v_{k}^{\left(u_{2}\right)}\\ \vdots & \vdots & \vdots & \vdots \\ v_{1}^{\left(u_{N}\right)} & v_{2}^{\left(u_{N}\right)} & \cdots & v_{k}^{\left(u_{N}\right)}\\ v_{1}^{\left(t\right)} & v_{2}^{\left(t\right)} & \cdots & v_{k}^{\left(t\right)} \end{array}\right]. \end{array}$$

Due to the uncertainty of the adjective language selection in the commentary library, the probability is used to describe its distribution characteristics. *P*_il_ is the probability of feature *a*_*i*_ values *v*_*l*_^(*u*_*i*_)^; after the commentator's emotional polarity is determined, the word is uncertain, and use the probability to eliminate the influences of the commenters' decision.

The uncertainty of the emotional polarity of comments is concentrated in the feature redundancy of the comment set. Mutual information can effectively measure the redundancy between variables in a feature set. It is thus possible to find a set of input features that has a large mutual information value with the identification category and low redundancy between other features. The feature Relation-Redundancy Coefficient (R^2^C) is used for discrimination considering both the range of feature values and the distribution of values.

In the feature selection process, the joint action of multiple candidate features on the category *c*, due to the redundancy. In this paper, the redundancy between *a*_*ƛ*_ and selected feature *S* and the redundancy between all features in *S* are collectively referred to as the redundancy of the feature, denoted by *M*(*c*; *a*_*ƛ*_; *S*). The eigenvalue number of feature *a*_*i*_ is *k*; then, its information entropy can be denoted as

(3) $$\begin{array}{c} E\left(a_{i}\right)=\overset{k}{\underset{l=1}{\sum }}P_{\text{il}}{\;\log}_{2}\frac{1}{P_{\text{il}}}. \end{array}$$

If *a*_*i*_ ∈ *A*, *a*_*j*_ ∈ *A*, and *a*_*j*_ ≠ *a*_*i*_, according to the joint distribution rate, the conditional entropy can be denoted as

(4) $$\begin{array}{c} E\left(a_{i}\;|\;a_{j}\right)=-\underset{a_{i},a_{j}\in A}{\sum }\underset{a_{j}\neq a_{i}}{\sum }P\left(a_{i},a_{j}\right)\log_{2}\;P\left(a_{i}\;|\;a_{j}\right). \end{array}$$

**Definition 1 .**

Comment space mutual information.

In Θ, the mutual information between *a*_*i*_ ∈ *A* and *a*_*j*_ ∈ *A* in feature set *A* can be expressed as

(5) $$\begin{array}{c} M\left(a_{i};a_{j}\right)=\underset{a_{i},a_{j}\in A}{\sum }\underset{a_{j}\neq a_{i}}{\sum }P\left(a_{i},a_{j}\right)\log_{2}\frac{P\left(a_{i}\;|\;a_{j}\right)}{P\left(a_{i}\right)P\left(a_{j}\right)}. \end{array}$$

The larger *M*(*a*_*i*_; *a*_*j*_) is, the closer the relationship between the feature random variables *a*_*i*_ and *a*_*j*_ is; when *M*(*a*_*i*_; *a*_*j*_) approaches zero, the two are independent of each other. The relationship between mutual information and information entropy can be expressed as

(6) $$\begin{array}{c} M\left(a_{i};a_{j}\right)=E\left(a_{i}\right)-E\left(a_{i}\;|\;a_{j}\right). \end{array}$$

### 3.2. Feature Selection Based on Mutual Information

**Definition 2 .**

Let *ℏ*_s_ be the ratio of the mutual information *M*(*c*; *a*_s_) between selected feature *a*_s_ and identification category *c* to the information entropy *E*(*a*_s_) of the feature *a*_s_; then, *ℏ*_s_=*M*(*c*; *a*_s_)/*E*(*a*_s_), 0 ≤ *ℏ*_s_ ≤ 1.

*ℏ*_s_ meets the following characteristics:

- When the range of feature values is the same, the more uniform the value is, the less important it is
- When the feature values are evenly distributed, the larger the value range is, the less important it is
- Then, the mutual information formula of feature redundancy in the MIFS-U method is expressed as

(7) $$\begin{array}{c} M\left(c;a_{ƛ};S\right)=\beta \underset{a_{\text{s}}\in S}{\sum }\left\{\hbar_{\text{s}}\cdot M\left(a_{\text{s}};a_{ƛ}\right)\right\}. \end{array}$$

The ratio of mutual information between maximum correlation and minimum redundancy denotes the ratio of feature correlation and redundancy. When ∑_*f*_s_∈*S*_{*ℏ*_s_ · *M*(*a*_s_; *a*_*ƛ*_)} > 0,

(8) $$\begin{array}{c} \vartheta =\frac{M\left(c;a_{ƛ}\right)}{\delta \sum_{a_{\text{s}}\in S}\left\{\hbar_{\text{s}}\cdot M\left(a_{s};a_{ƛ}\right)\right\}}, \end{array}$$

*δ* is a constant used to measure the influence degree of redundancy between features in the feature set on classification accuracy, and it can be set according to the actual situation. The parameter called the feature Relation-Redundancy Coefficient (R^2^C) that measures the redundancy of the selected feature set is expressed by a nonnegative number *ℜ*:

(9) $$\begin{array}{c} ℜ=\left\{\begin{array}{cc} \infty , & \underset{a_{\text{s}}\in S}{\sum }\left\{\phi_{\text{s}}\cdot M\left(a_{\text{s}};a_{ƛ}\right)\right\}=0\;\mathrm{or}\;S=\emptyset ,\\ \vartheta , & \text{others}. \end{array}\right. \end{array}$$

In Θ, the Relation-Redundancy Coefficient has the following four effects:

- When *ℜ*=0, the correlation of candidate features *a*_*ƛ*_ and the identification category *c* is zero; *a*_*ƛ*_ is an irrelevant feature of Θ.
- When 0 < *ℜ* < 1, the redundancy of the candidate features *a*_*ƛ*_ and *a*_*s*_ is stronger than *a*_*ƛ*_ and other features; then, it is a redundancy feature.
- When *ℜ* > 1, the correlation between the candidate feature *a*_*ƛ*_ and the identification category *c* is stronger than the redundancy of the candidate features *a*_*ƛ*_ and *a*_s_ and brings new information for classification; then, it is called the correlation feature. Here we set a threshold *θ*(*θ* > 1) based on the actual values for the correlation features. The features are strong correlation features when *ℜ* ≥ *θ*; otherwise, they are weak correlation features.
- When *ℜ*=*∞*, it only needs to analyze the mutual information *M*(*c*; *a*_*ƛ*_) between *a*_*ƛ*_ and the identification category *c*; the corresponding *a*_*ƛ*_ of the maximum value *ℜ* can be selected into the set *S*. According to the abovementioned effects, the optimal feature set *S*={*a*_*j*_ | *j*=1,2,…, *φ*} including the *φ* features is finally obtained.

Given the mutual information and redundancy of the features, the empirical index *α* is given by the expert. Using the mutual information method to obtain the comprehensive weight *w*_*j*_ of the feature *a*_*j*_ in the comment space Θ,

(10) $$\begin{array}{c} w_{j}=\alpha \times \frac{M\left(c;a_{ƛ};S\right)}{\sum_{a_{ƛ}\in S}M\left(c;a_{ƛ};S\right)}+\left(1-\alpha \right)\frac{M\left(c;a_{j}\right)}{\sum_{a_{j}\in S}M\left(c;a_{j}\right)}. \end{array}$$

As an important parameter of the model, *w*_*j*_ plays an important role in the accuracy of the classification.

### 3.3. Emotional Polarity Selection Based on Mutual Information Weight

Based on the corpus data in the basic database, we obtain the optimal subset with the least feature redundancy and the relative weights of each feature in the feature set and calculate the emotion value of the unmarked corpus in the marked feature based on this weight. The specific steps are as follows:

- Extract the emotional words from unmarked corpus and convert them to a basic corpus.
- According to the basic corpus, the optimal features including weights that remove redundant features are filtered out.
- The eigenvalues corresponding to the feature nouns are assigned according to the emotional dictionary NTUSD compiled by Taiwan University; the positive word is assigned 1, the negative word is assigned −1, and the emotional value according to the weight is calculated (which ignores the impact of the adverb or grammatical structure for the emotional value) and the emotional threshold based on the basic corpus is set.
- Judge the polarity and accuracy of the test corpus according to the weights and emotional thresholds based on the training library.

## 4. Numerical Experiment

Our experimental analysis is performed between mutual information method and emotion lexicon, TI-IDF and SVM. Using four datasets crawled from the Haodf's online platform to evaluate the performance of our proposed ALEIM method, the experiment is divided into four aspects:

- The datasets used in the experiments.
- The overall flow and evaluation measures of the experiments.
- The description of the experimental details by using the four datasets crawled from the Haodf's online platform.
- The discussion of the experiments.

### 4.1. Datasets

**Figure 1:**
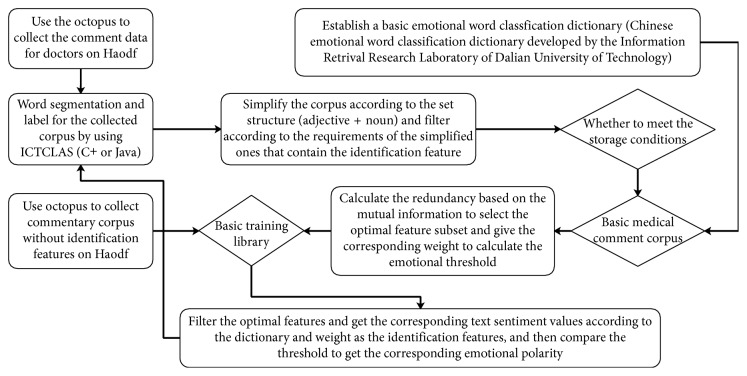
The preparation process of experimental datasets in this paper.

The experimental datasets are crawled from the Haodf's online platform. These medical service comments are extracted using the octopus, and then the word segmentation is reorganized using Java programming, and each sentence in the comment is split into the metamatrix structure of “noun + verb.” We first select 100 doctors and randomly collect 750 data in their comment areas and construct four basic corpus training libraries with different comments based on the above data, which is shown in Figure 1.

The number of positive and negative comments in the four basic corpus training libraries varies, and the positive comments ratio is higher than the negative ones. Due to the random extraction of the comment data as a corpus training library, the distribution of positive and negative comments in the training library is uncertain. Such randomly extracted data are used as training corpus data, which can test not only the dependence of different classification algorithms based on different category numbers but also the learning ability of the specific marked category based on a small sample. 400 data are prepared as the test data in Table 2, including 200 positive comments and 200 negative comments to test the accuracy of the training library for emotion classification under different algorithms.

When feature extraction is performed, the features extracted from the corpus with 100 data are all included in the other volumes corpus; the features extracted from the corpus with 150 data are all included in the corpus of 200, 300, and 400 data; the corpuses of 200 and 300 data extract the same features; the feature number extracted from the test corpus with 400 data is 42, and an additional feature is extracted from the corpus with the 200 and 300 data.

Since the data are randomly crawled, the corpus has low data repeatability between each other, so it can approximate that the probability of new features appearing decreases rapidly as the selected comment corpus data increase. Therefore, the amount of comment data for a suitable training corpus is determined, and the extracted features can contain almost all the features included in the medical comments (some special features extracted by small probability often not related to the medical service itself). This indicates that the features in comments often have limitations compared with traditional commodity comments due to the uniformity and standardization of medical services. The general commodity comments are not fixed due to the feature attributes of products; the products are highly different, and different products often contain unique features, which often affect the overall polarity of the comments. Therefore, commodity comments have high requirements for feature extraction, and it is necessary to continuously update the extracted features based on a large amount of data to achieve accurate classification of emotional polarity. Since the medical service does not have the variability of general commodity, the features of the comments are limited, so selecting a certain amount of data extract features can almost involve all the features in the medical service comments.

### 4.2. Experimental Design and Evaluation Measures

**Figure 2:**
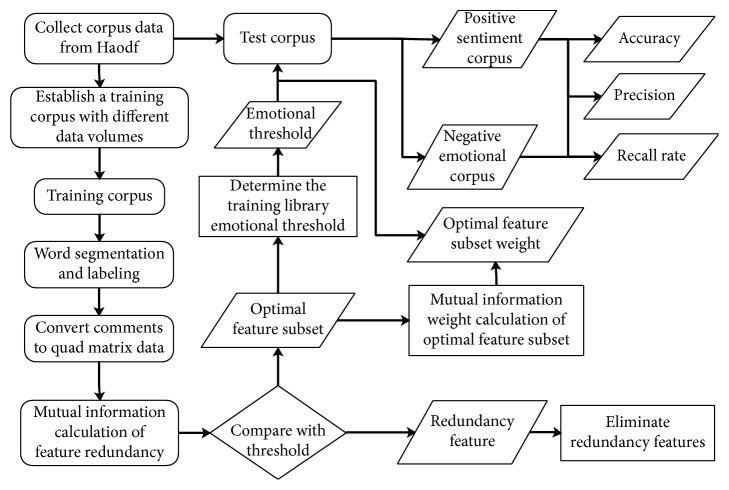
The overall flowchart of experiments in this paper.

We employ Taiwan University NTUSD Simplified Chinese Emotion Dictionary Corpora for emotion and emotion classification. The overall flowchart of experiments in this paper is shown in Figure 2.

The SVM and feature weight algorithm used in this experiment are implemented by using MATLAB. Among them, the mutual information algorithm and the IDF algorithm calculate the feature weight by using the basic corpus and then combine the emotion dictionary NTUSD to calculate the emotion value of the corpus in the training library and set the emotional threshold according to the corpus data (calculate the positive and negative comments, respectively, and then use the weighted average of the two types emotional mean as the emotional threshold). The emotional polarity of the test corpus based on the feature weight and the threshold is judged. We have selected the following indicators as evaluation indicators:

- True positive: originally positive emotions, classified as positive emotions.
- True negative: originally negative emotions, classified as negative emotions.
- False positive: originally negative emotion, classified as positive emotion.
- False negative: originally positive emotion, classified as negative emotion.

The accuracy reflects the ability of the classifier to determine the entire sample: the positive decision can be positive and the negative decision negative and can be expressed as

(11) $$\begin{array}{c} A=\frac{\left(\text{TP}+\text{TN}\right)}{\left(\text{TP}+\text{FN}+\text{FP}+\text{TN}\right)}. \end{array}$$

The precision reflects the proportion of the true positive sample in the positive case determined by the classifier and can be expressed as

(12) $$\begin{array}{c} P=\frac{\text{TP}}{\left(\text{TP}+\text{FP}\right)}. \end{array}$$

The recall reflects the proportion of positive cases that are correctly judged as the total positive examples and can be expressed as

(13) $$\begin{array}{c} R=\frac{\text{TP}}{\left(\text{TP}+\text{FN}\right)}. \end{array}$$

### 4.3. Implementation Details of Experiments

**Figure 3:**
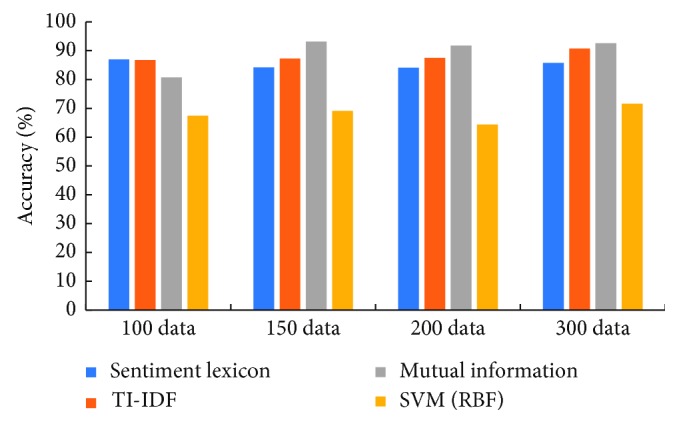
The accuracy of the four methods used in this paper.

Figure 3 shows that the accuracy of the classification algorithm of IDF and mutual information considering the feature weight and the emotion dictionary-based classification algorithm are significantly higher than the SVM algorithm using the Gaussian kernel function for four basic corpus libraries. As the number of samples increases, the accuracy of emotion lexicon maintains constant basically. However, as the number of samples increases, the accuracy of mutual information increases rapidly and is higher than the other three methods. As can be seen from Figure 3, the performance of mutual information method is better than the other three methods. SVM algorithm requires that the number of different types in the training database must be substantially the same to achieve optimal learning. However, the online medical service comments have a large proportion of positive and negative polarities; support vector machine algorithm is difficult to achieve the optimal data ratio. Constructing the training library according to the actual ratio often leads to the identification of negative polarity data with less proportion and leads to lower overall accuracy.

Table 3 illustrates the detailed significant test results of accuracy between mutual information and other three methods in terms of the *p* value on the four basic corpus libraries. As can be seen from the table, the mutual information method is superior to the other three methods on 150 data, 200 data, and 300 data. The results show that when the sample size increases, *p* values between mutual information and other three methods are less than 0.05. This means the classification results of mutual information method are significantly better than the other three methods.

**Figure 4:**
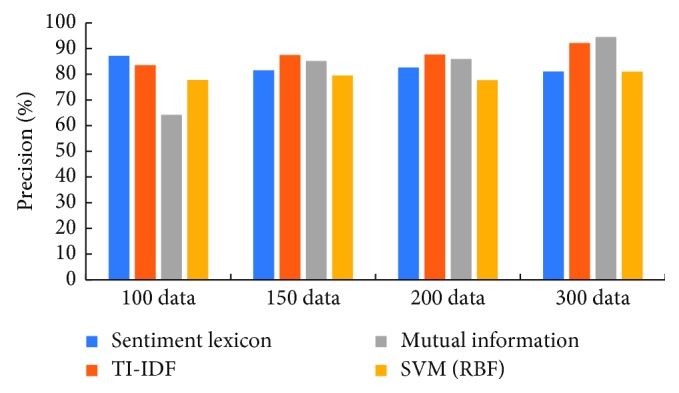
The precision of the four methods used in this paper.

Figure 4 shows that the precision of the classification algorithms of IDF and mutual information considering the feature weight are slightly higher than the other two algorithms. The mutual information algorithm has lower precision when the training data are less, and the precision is improved with the training data increase but is slightly lower than the IDF weighting algorithm.

Table 4 illustrates the detailed significant test results of precision between mutual information and other three methods in terms of the *p* value on the four basic corpus libraries. As can be seen from the table, there is a significant difference among the mutual information method, emotion lexicon, and SVM methods because *p* values between mutual information and other two methods are less than 0.05, but when the number of samples increases, there is no significant difference between the mutual information method and TI-IDF method.

**Figure 5:**
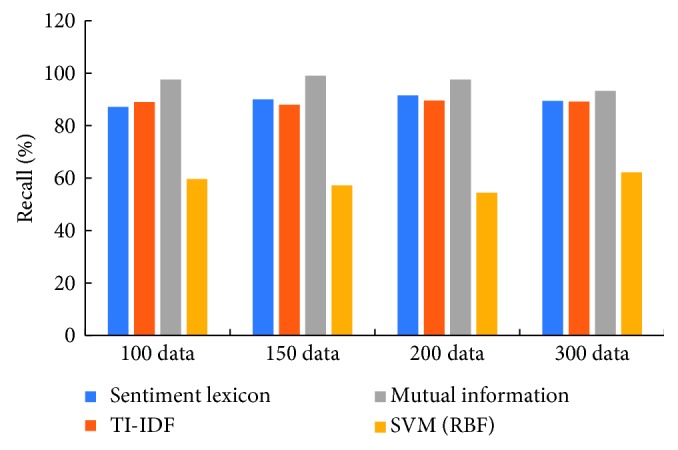
The recall of the four methods used in this paper.

Figure 5 indicates that our proposed algorithm which considers the weight of each feature has the superior performance than other two comparison approaches. Since the negative emotion polarity data of the training inventory are less, the recall of the other two algorithms is extremely low, and the weight of the feature weight algorithm is not dependent on the weight of the data category, so the learning effect on the limited negative polarity data is better, and the recognition of the negative emotion data in the test data is higher. The recall rate of the mutual information algorithm is significantly higher than that of the IDF algorithm. It shows that the mutual information algorithm considering the feature weight has strong recognition ability for negative emotion.

Table 5 illustrates the detailed significant test results of recall between mutual information and other three methods in terms of the *p* value on the four basic corpus libraries. From this table, it can be seen that there is a significant difference among the mutual information method, emotion lexicon, and SVM methods because *p* values between mutual information and other two methods are less than 0.05, but there is no significant difference between the mutual information method and TI-IDF method.

**Figure 6:**
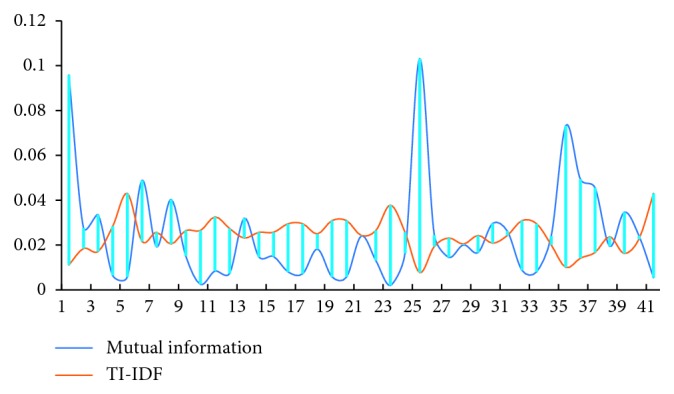
The difference between mutual information algorithm and TI-IDF algorithm.

Figure 6 shows the comparison of 41 feature weights in 300 training library corpora under mutual information weighting algorithm and TI-IDF algorithm. It can be seen that the weights of the two algorithms of feature 1, feature 25, feature 35, and feature 5 and feature 41 are quite different, corresponding to the condition, attitude, doctor, and side effects and consultation.

The mutual information algorithm weights are significantly higher than the IDF weights for the first three features. These three features are commonly found in medical comments. IDF algorithm believes that these comments with high frequency are of lower importance and are filtered to give small weights, while mutual information algorithm according to the high mutual information value and low redundancy of the identification feature gives the high weight, and such weight causes the mutual information algorithm to have a lower accuracy than the IDF in identifying the positive emotional polarity. These features are used as the basic features of comments; it tends to have a lower guiding effect on the emotional polarity of the reviewer in the positive comments and a primary role in the orientation of the emotional polarity in the negative comments.

In the latter two features, the mutual information algorithm weight is significantly lower than IDF algorithm. These features belong to the low-frequency features and appear 6 times and 7 times in 300 data, respectively. The IDF algorithm assumes that low-frequency words can more affect the emotional polarity of the comments for the comment library as a whole, and the mutual information algorithm considers these features to be small mutual information values with high redundancy and low weight. The experiments show that these two features actually weaken the emotional polarity of the comments. The IDF algorithm classifies all the errors in the six test comment categories with the above two features, and the mutual information recognition rate is 100%.

### 4.4. Discussion of Experiments

From the above experimental analysis, we can obtain that mutual information is the most appropriate method to solve such problem. It shows good performance in terms of accuracy when the number of samples increases and only requires a moderate computational cost for solving emotion classification problems of short texts for online medical knowledge sharing community. However, in terms of precision and recall, there is no significant difference between the mutual information method and TI-IDF method, but Figure 6 shows that the accuracy of IDF algorithm in identifying negative emotion polarity is significantly lower than that of mutual information algorithm. Experiments show that low-frequency words existing in medical review data are often redundant features, and mutual information algorithm has higher accuracy for the identification of such redundant features. However, our experiments need to be further improved due to only four basic corpus libraries involved in the experiment. Therefore, we plan to crawl more different types comments on online medical knowledge sharing community to achieve parameter optimization and method performance promotion.

## 5. Conclusions

Emotion analysis has been widely used in many fields and becomes an important tool for extracting emotional information of the comments. Emotional analysis in medical knowledge sharing community is still relatively lacking compared with the general commodities. The information recipients in the medical knowledge sharing community are more concerned with the intensity of the emotional words in the comments or the overall evaluation. In this research, we propose an adaptive learning emotion identification method based on mutual information feature weight, which captures the correlation and redundancy of features. Its effectiveness is verified on the dataset crawled from the Haodf's online platform, and we employ Taiwan University NTUSD Simplified Chinese Emotion Dictionary Corpora for emotion classification. Finally, the experimental results show that the proposed ALEIM method can achieve good performance, especially in terms of the low-frequency words feature extraction in comments of the online medical knowledge sharing community.

## Figures and Tables

**Table 1:** Summary of typical previous studies for the emotion analysis challenges.

Author	Year	Domain oriented	Challenge type	Review structure
Jia et al. [3]	2009	Health/medical domain	Theoretical	Semi-structured
Hogenboom et al. [4]	2011	Movie reviews	Theoretical	Unstructured
Alexandra and Ralf [5]	2009	Online news reviews	Theoretical	Semistructured/unstructured
Mukherjee and Bhattacharyya [6]	2012	Products	Technical	Semistructured
Chetan and Atul [7]	2014	Tweets	Technical	Unstructured
Doaa and Osama [8]	2015	Scientific papers	Theoretical + technical	Structured

**Table 2:** The test data for emotion classification under different algorithms.

The number of data	Positive	Negative	The number of feature	Used for
100	70	30	37	Training corpus
150	100	50	39	Training corpus
200	120	80	41	Training corpus
300	180	120	41	Training corpus
400	200	200	42	Test corpus

**Table 3:** The detailed significant test results of accuracy between MI and other methods.

Datasets	Metrics	Methods		
		MI and emotion lexicon	MI and TI-IDF	MI and SVM (RBF)
100 data	*p* value	0.0906	0.0063	0.1304
150 data		0.0487	0.0197	0.0043
200 data		0.0435	0.0437	0.0226
300 data		0.0255	0.0432	0.0021

**Table 4:** The detailed significant test results of precision between MI and other methods.

Datasets	Metrics	Methods		
		MI and emotion lexicon	MI and TI-IDF	MI and SVM (RBF)
100 data	*p* value	0.0413	0.0043	0.0343
150 data		0.0387	0.0667	0.0342
200 data		0.0234	0.0731	0.0106
300 data		0.0055	0.0902	0.0049

**Table 5:** The detailed significant test results of recall between MI and other methods.

Datasets	Metrics	Methods		
		MI and emotion lexicon	MI and TI-IDF	MI and SVM (RBF)
100 data	*p* value	0.0313	0.1025	0.0034
150 data		0.0478	0.0706	0.0147
200 data		0.0443	0.0831	0.0321
300 data		0.0142	0.0502	0.0079

## Data Availability

The experimental data come from the Haodf's online platform and can be crawled from https://www.haodf.com.
